# Supplementary material for: A genomic surveillance framework and genotyping tool for Klebsiella pneumoniae and its related species complex
Source: Nat Commun. 2021 Jul 7;12:4188. doi: 10.1038/s41467-021-24448-3 (PMC8263825; doi:10.1038/s41467-021-24448-3)
Supplement: Supplementary file 1 — Supplementary information [file 41467_2021_24448_MOESM1_ESM.pdf]

Supplementary Information

Supplementary Notes

Supplementary note 1: Species and subspecies assignment

To prepare the taxonomy for Kleborate's species identification, we ran Bacsort (github.com/rrwick/Bacsort) on all available Enterobacterales genomes in RefSeq as of March 2019. Particular attention was paid to *Klebsiella* species and subspecies definitions, using recent publications as a guide[1–4]. Subspecies distinctions were only made for the KpSC, while all other taxa were categorized to a species level. In order to define a reference set of genomes that were representative of diverse phylogenetic clusters, Bacsort was run twice: once with a Mash distance threshold of 0.005 and again with a Mash distance threshold of 0.01. The lower-threshold run resulted in 4875 representative genomes while the higher-threshold run resulted in 1875 representative genomes. We kept the lower-threshold (higher-resolution) cluster representatives for *Klebsiella* and *Raoultella*, and the higher-threshold (lower-resolution) cluster representatives for all other genera, resulting in a total of 2619 reference genomes, and built a Mash database of these (sketch size of 1000). A copy of this database is available in the Kleborate repository (/data directory), a FastME[5] tree of all pairwise Mash distances is available at https://www.doi.org/10.6084/m9.figshare.13372613 and a summary tree depicting the reference taxonomy is shown in Supplementary Figure 1a-b. To assign taxonomy to a query genome, Kleborate calculates pairwise Mash distances between the query assembly and all reference genomes, and finds the closest match (smallest distance). If this nearest-neighbour Mash distance is $\leq$0.02, Kleborate reports the species/subspecies as a strong match (defined from inspection of the empirical distribution, Supplementary Figure 1c-d). If the distance is >0.02 and $\leq$0.04, Kleborate reports the species/subspecies as a weak match. If the distance is >0.04, we consider this too distant from any of the reference genomes and therefore cannot assign a species (in which case Kleborate reports the species as 'unknown'). Note, the subspecies *K. pneumoniae* subsp.

rhinoscleromatis and *ozaenae* each form a monophyletic lineage within *K. pneumoniae sensu stricto*[6] and are thus first identified as species *K. pneumoniae* by Mash distance, and subsequently identified as subspecies on the basis of MLST.

We compared this approach with the well-established method of taxonomic assignment based on analysis of reads using Kraken2. We built a custom Kraken (v2.1.1) database using the same curated set of 2619 reference genomes, and used a set of *n*=285 diverse clinical isolates of Gram negative bacteria to compare the results of two different approaches to species identification: Mash distance-based identification of assemblies (species assigned based on nearest neighbour) and Kraken2-based identification of reads (species assigned based on most common taxon). For 283/285 isolates, both approaches gave identical results; the remaining two cases were mixed samples each containing two genomes (Supplementary Data 1). Sequence-based identification agreed with the original MALDI-TOF identification of the isolates in the majority of cases. A total of 259 isolates were identified as Enterobacterales by MALDI-TOF. Kleborate identified the same species as MALDI-TOF for 208 samples (80%); for a further 44 (17%) Kleborate identified sequences for species that were underrepresented, mis-labelled or missing from the MALDI-TOF database (*K. variicola, K. quasipneumoniae*, *Citrobacter portucalensis, Escherichia marmotae, Enterobacter hormachei*), in line with previous reports[7]; for 3 samples the sequence data was mixed/contaminated; and the 4 remaining cases appear to be mis-identification by MALDI-TOF, as the sequence data for these isolates showed no signs of contamination and were unambiguously and consistently identified using both sequence-based methods.

Supplementary note 2: Genotyping of clinical isolates from EuSCAPE surveillance study

In the original study[8], the following approaches were used to infer genotypes: (i) Mash v2.0 with the RefSeq bacterial database was used to separately assign raw sequence reads and assemblies to species, (ii) the MLST calling function of ARIBA v2.6.1 with the *K. pneumoniae* MLST database from PubMLST used to assign STs, (iii) Kaptive v0.5.1 used to assign a K locus, and (iv) ARIBA v2.6.1 with a custom resistance gene database used to identify genes conferring resistance to carbapenems and extended-spectrum ß-lactams, in addition to the *ompK35* and *ompK36* porin genes followed by a manual check for the completeness of the sequence. While the presence or absence of each of virulence loci was also included in the supplementary data output of the original paper, no details were provided on how this information was derived.

The concordance between the genotypes and/or presence of AMR and virulence loci reported in the original EuSCAPE study and those reported by Kleborate was high (81.6-100%, Supplementary Data 3). Notably, there was 100% concordance for species assignment, ≥99% for the detection of the *ybt, iro, iuc* and *rmpA* virulence loci and 98.7% for the *rmpA2* locus. There were 99 ST discrepancies, all representing genomes that were not assigned STs in the original study but were assigned to recently described STs by Kleborate (i.e. STs that have been defined since the original study was published). Despite using the same approach for K-locus assignment, there was a single inconsistency that was also attributed to a database update, wherein Kleborate identified the best matching locus as KL163 which was not present in the version of the database used in the original EuSCAPE analysis (note that the original Kaptive match confidence was not reported in the original paper).

Carbapenemase genes were detected with ≥99.5% concordance, and Omp mutations with ≥98.6% (excluding those types of mutations reported by Kleborate that were not explored in the original study i.e. OmpK36 GD and TD insertions). However, ESBL gene detection showed lower concordance (88.6%). The majority of these differences likely result from the use of different databases or database versions, which is well known to cause inconsistencies for AMR genotyping[9]. Notably, Kleborate uses a curated version of the CARD AMR database, wherein the classification of SHV ß-lactamase alleles has been updated to reflect recent advances in understanding the sequence variations that result in true changes to the β-lactamase phenotype[10] (see below and Supplementary Data 12). As a result, the SHV-16, SHV-27, SHV-28, SHV-30, SHV-38, SHV-40, SHV-41, SHV-42, SHV-99, SHV-100 alleles classified as ESBL genes in the original EuSCAPE paper are not counted as ESBL genes by Kleborate (accounts for 63.5% of ESBL discrepancies).

Supplementary note 3: Differentiation of intrinsic and acquired SHV β-lactamases

β-lactamases, including the SHV enzymes, are further divided into subclasses (e.g. extended-spectrum, carbapenem-hydrolyzing) based on their ability to hydrolyze different substrates (e.g. third generation cephaloporins, carbapenems). A subset of allelic variants have been subjected to biochemical and phenotypic tests that confirm their subclass assignments, but many of the more recently identified alleles have not been subjected to the same analyses, and their predicted subclass assignments differ between databases. In *K. pneumoniae*, the attribution of activity to SHV enzymes is particularly confusing since nearly all genomes carry a 'wildtype' chromosomal SHV allele conferring narrow spectrum resistance (i.e. to ampicillin and earlier generation cephalosporins), but some carry additional mobile variants of SHV or other enzymes harbouring substitutions that confer ESBL activity. This has resulted in some chromosomal variants with wildtype (i.e. narrow) activity being erroneously assigned as ESBL in some reports and databases (e.g. SHV-27[11]). It also means that correct

identification of the precise allele/s present is important, as the mere presence of a SHV gene in *K. pneumoniae* does not imply ESBL (whereas it typically does in other species which do not have intrinsic wildtype SHV).

To make Kleborate's output more informative and accurate in terms of the SHV alleles and spectrum of activity reported in *K. pneumoniae* genomes, we curated the SHV alleles present in the CARD database, reviewing the class assignment and supporting evidence for each allele. A recent study used systematic mutagenesis and phenotyping to test which specific substitutions in naturally-occurring SHV alleles confer resistance to β-lactamase inhibitors and extended-spectrum cephalosporins[10] (summarised in Supplementary Data 11). For example, substitutions at Ambler positions 179 or 238 were sufficient to confer the ESBL phenotype, while those at position 69 were confirmed to confer resistance to β-lactamase inhibitors. We therefore began by re-assigning all SHV alleles on the basis of the presence/absence of these confirmed functional mutations (noted as class modifying in Supplementary Data 11). In most cases (>80%) this assignment matched that in the Beta-lactamase Database (BLDB, http://www.bldb.eu/ which supersedes the Lahey database). Where there was a discrepancy vs. BLDB, we reviewed the evidence in the published literature (based on references cited in BLDB, and searches for the allele name in PubMed and NCBI). This confirmed 4 additional alleles with a proven modified spectrum of activity, for which we updated the assignment in our database (SHV-16[12], SHV-57[13], SHV-70[14] as ESBL; SHV-31[15] as inhibitor resistant); the remainder we reported to BLDB as likely errors (full details in Supplementary Data 12). The final set of SHV allele assignments is given in Supplementary Data 12.

Kleborate reports alleles recorded as 'wildtype' in the 'Bla_chr' column, as current data indicates that non-ESBL non-inhibitor resistant forms are all chromosomally encoded; other alleles are reported in the relevant functional column and are included in 'acquired genes'

and ‘acquired resistance’ counts. In addition, Kleborate searches for the specific SHV mutations in Supplementary Data 11 and reports these in the ‘SHV_mutations’ column. To accommodate the possibility of novel alleles with modified activity, if one of the mutations noted as ‘class modifying’ is found but the closest matching allele is assigned as ‘wildtype’, the allele will be recorded in the relevant functional column. To accommodate the possibility that a novel wildtype variant happens to be closest to a variant with modified activity, if none of the mutations in Supplementary Data 11 are identified then the allele will be recorded in the ‘Bla_chr’ column.

Supplementary Figures

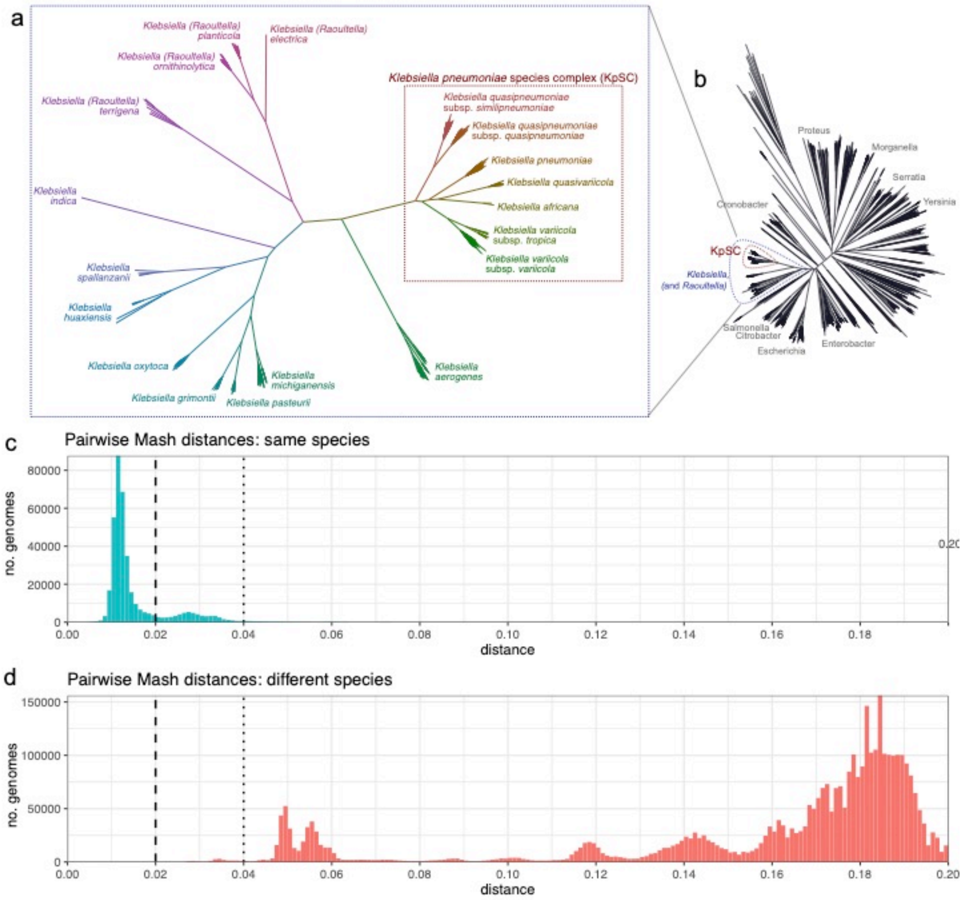

Supplementary Figure 1. Overview of Kleborate species reference database.

Phylogenetic relationships between genomes belonging to species of (a) *Klebsiella* (and *Raoultella*), and (b) Enterobacterales included in Kleborate’s species database. The tree was

inferred from mash distances of representative genomes (see Supplementary Notes). Distribution of pairwise Mash distances between genomes belonging to (c) same and (d) different *Klebsiella* species/subspecies. The number of genomes is shown on the y-axis and distance on the x-axis. Kleborate thresholds for strong and weak matches are indicated by the dashed and dotted lines respectively.

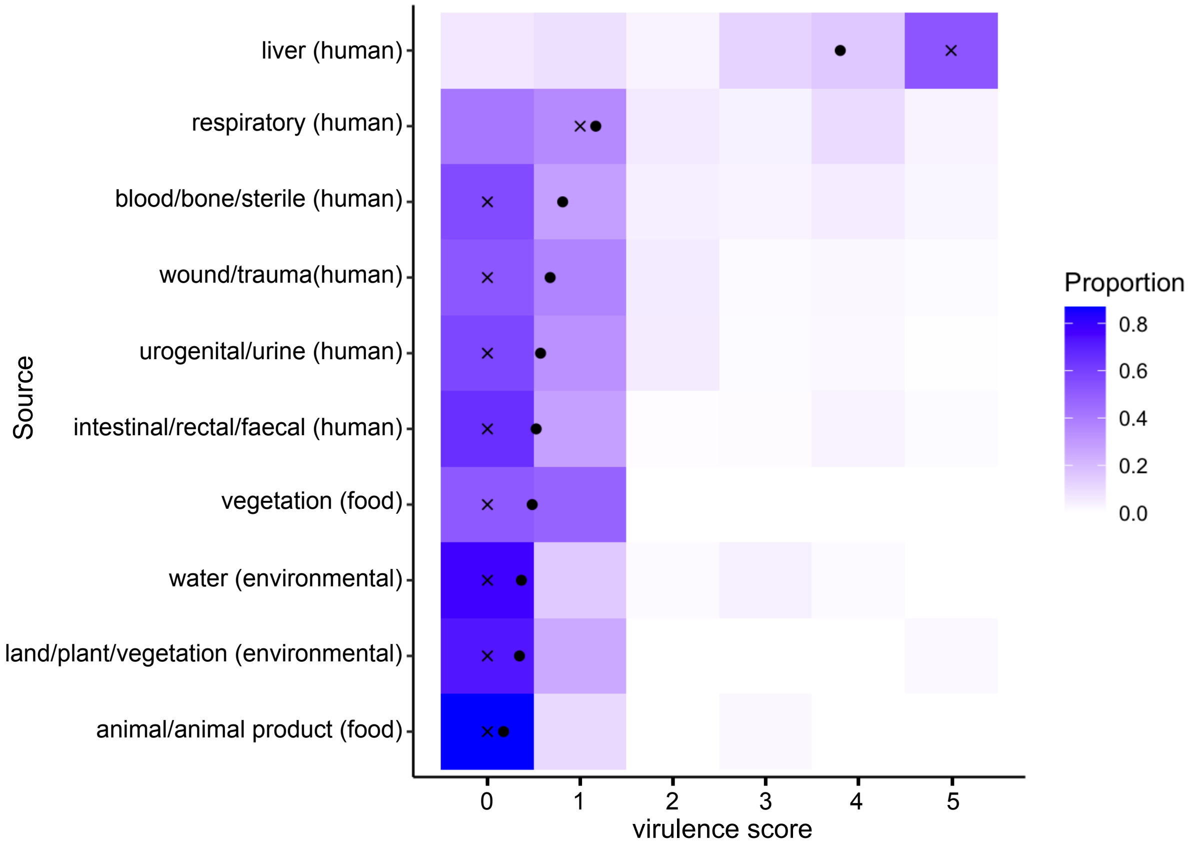

Supplementary Figure 2. Distribution of virulence scores for isolates across different sampling sources. The shading represents the proportion of isolates within a particular sampling source (row) assigned to each virulence score (labelled in the x-axis). Circles indicate mean score per source, crosses indicate median score per source.

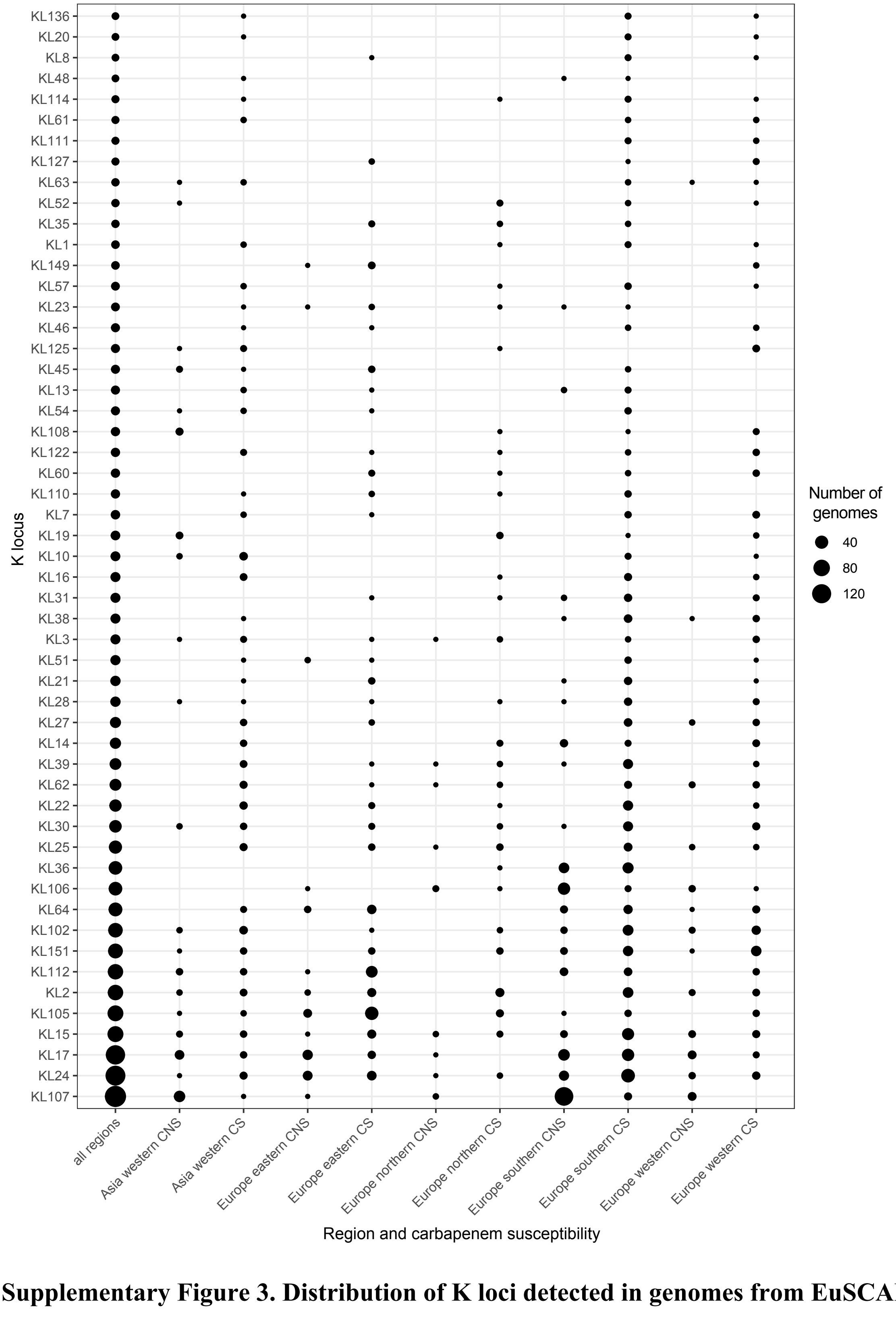

Supplementary Figure 3. Distribution of K loci detected in genomes from EuSCAPE study. The overall prevalence of each K locus (KL) is shown in the first column (i.e. all regions), ordered by prevalence, followed by detection in each geographical region and

carbapenem susceptibility: CNS – carbapenem non-susceptibility, CS – carbapenem susceptibility.

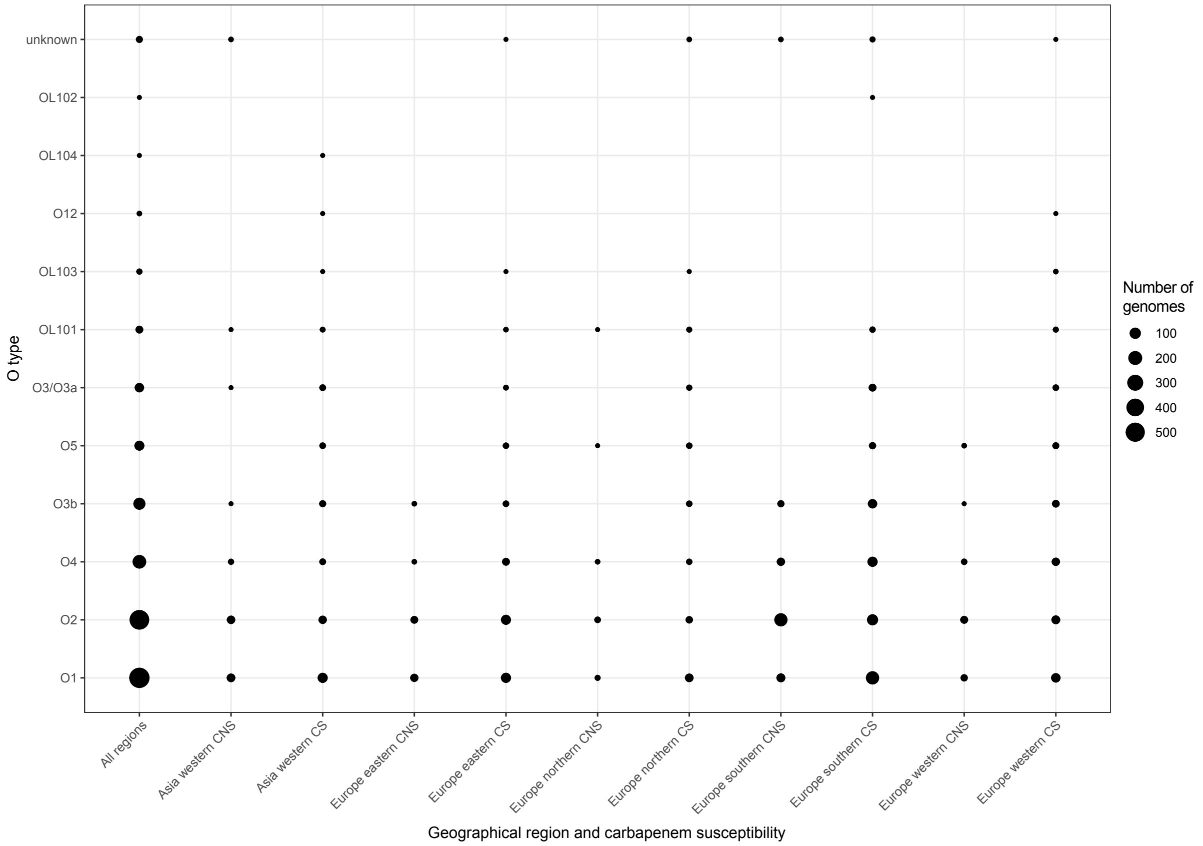

Supplementary Figure 4. Distribution of O types detected in genomes from EuSCAPE study ordered by prevalence. The overall prevalence of each O type is shown in the first column (i.e. all regions), ordered by prevalence, followed by detection in each geographical region and carbapenem susceptibility: CNS – carbapenem non-susceptibility, CS – carbapenem susceptibility.

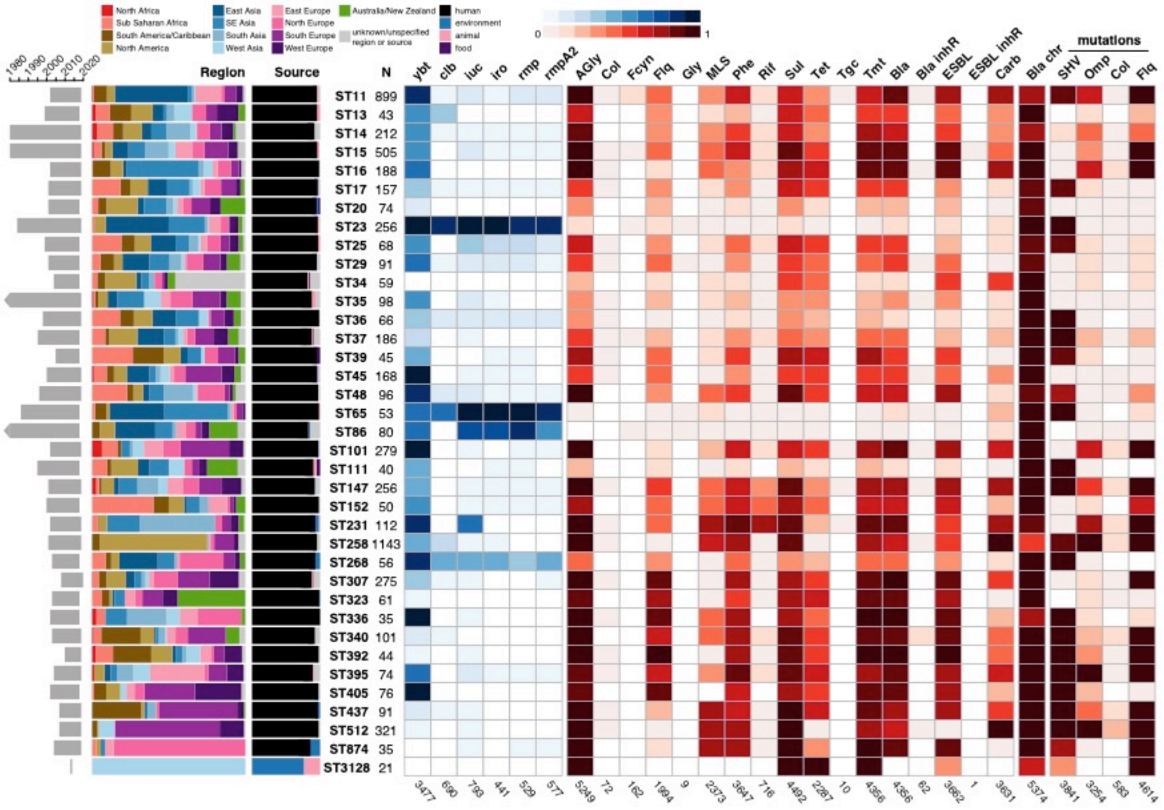

Supplementary Figure 5. Summary of isolate collection metadata and results of Kleborate virulence and resistance genotyping, for *K. pneumoniae* non-redundant genomes belonging to the 30 most common lineages. From left to right: barplots showing source information by geographical region and sample type (coloured as per inset legend); heatmaps showing prevalence of virulence loci (blue) and predicted AMR drug classes (red) (as per inset scale bars). Genomes are summarised by species, ordered by species complex: KpSC, *K. pneumoniae* species complex; KoSC, *K. oxytoca* species complex; and other *Klebsiella*. In the heatmaps, the total number of genomes in which each type of virulence/AMR determinant was detected are indicated below each column. Column names are as follows: ybt, yersiniabactin; clb, colibactin; iuc, aerobactin; iro, salmochelin; rmp, hypermucoidy Rmp; rmpA2, hypermucoidy rmpA2; AGly, aminoglycosides; Col, colistin; Fcyn, fosfomycin; Flq, fluoroquinolone; Gly, glycopeptide; MLS, macrolides; Phe, phenicols; Rif, rifampin; Sul, sulfonamides; Tet, tetracyclines; Tgc, tigecycline; Tmt, trimethoprim; Bla, β-lactamases; inhR, β-lactamase inhibitor; ESBL, extended-spectrum β-

lactamases; ESBL_inhR, extended-spectrum β-lactamase with resistance to β-lactamase inhibitors; Carb, carbapenemase; Bla_chr, intrinsic chromosomal β-lactamase; SHV, mutations in SHV; Omp, truncations/mutations in *ompK35/ompK36*; Col, truncations in *mgrB/pmrB* conferring colistin resistance; Flq, mutations in *gyrA/parC* conferring resistance to fluoroquinolones.

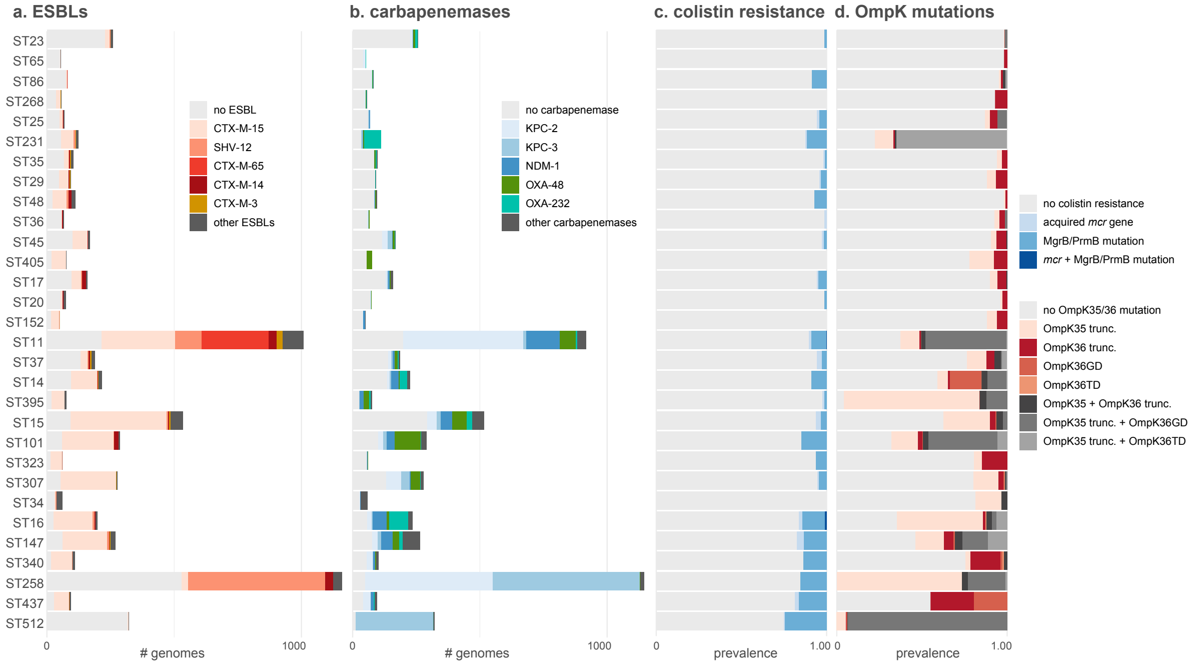

Supplementary Figure 6. Frequency of specific AMR genes and mutations identified in *K. pneumoniae* genomes. Barplots summarise frequencies of specific (a) extended-spectrum β-lactamase (ESBL) genes, (b) carbapenemase genes, (c) colistin resistance determinants, and (d) OmpK porin mutations identified by Kleborate in each of the top 30 most common *K. pneumoniae* STs from the non-redundant set of 9,705 publicly available *K. pneumoniae* genomes (see Methods, Supplementary Data 2). Trunc, truncation.

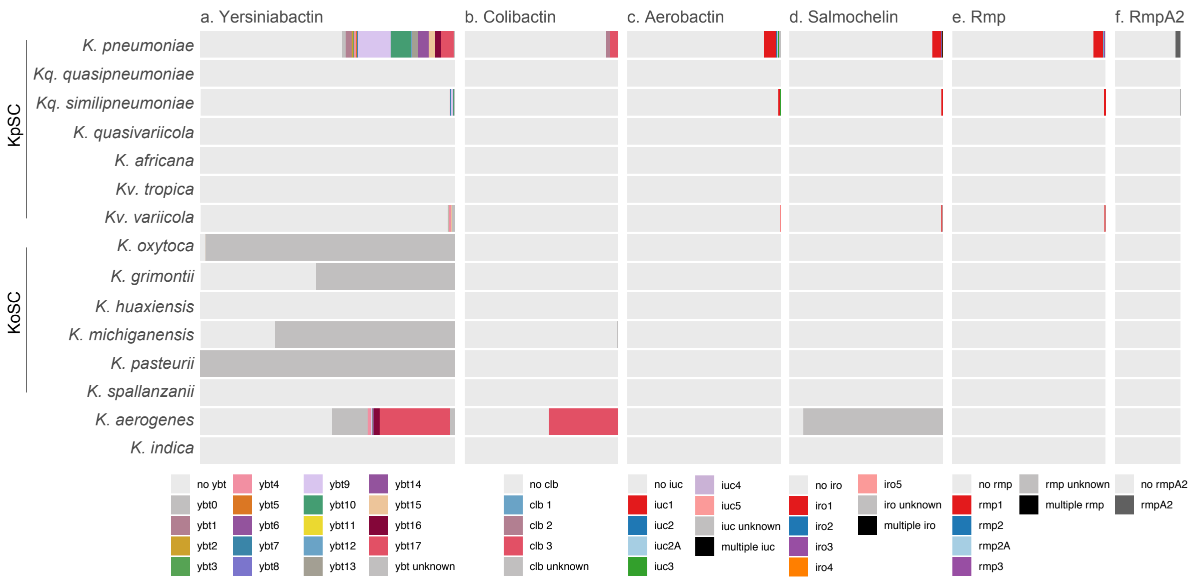

Supplementary Figure 7. Prevalence and breakdown of virulence loci by *Klebsiella* species. Data shown summarise Kleborate results for all 13,156 *Klebsiella* genomes publicly available as at July 17, 2020 (Supplementary Data 2). The scaled distribution of lineages is shown for each of (a) yersiniabactin (ybt), (b) colibactin (clb), (c) aerobacin (iuc), (d) salmochelin (iro) and (e) Rmp, followed by (f) presence/absence of RmpA2 coloured as indicated in the legend below each panel. Kq, *K. quasipneumoniae*; Kv, *K. variicola*; KpSC, *K. pneumoniae* species complex; KoSC, *K. oxytoca* species complex.

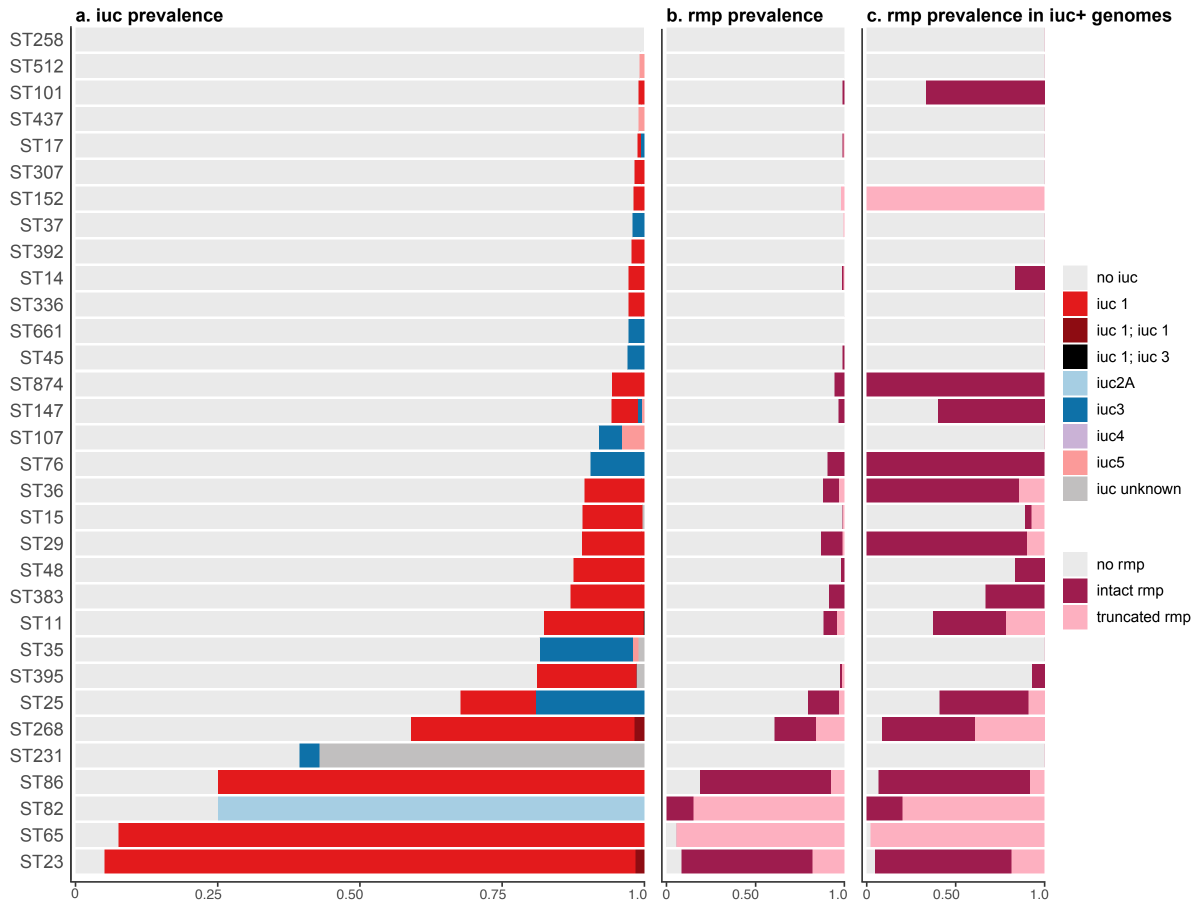

Supplementary Figure 8. Prevalence of aerobactin-encoding *iuc* and *rmp* hypermucoidy loci for the 30 most common *K. pneumoniae* lineages. Data summarise Kleborate results for each of the top 30 most common *K. pneumoniae* STs from the non-redundant set of 9,705 publicly available *K. pneumoniae* genomes (see Methods, Supplementary Data 2). Lineages were defined on the basis of multi-locus sequence types (STs). (a) Prevalence of *iuc*, colored by lineage(s) as per inset legend. (b) Overall prevalence of *rmp* and (c) prevalence of *rmp* among *iuc*+ genomes, colored by whether the locus is intact (i.e. no truncations detected in *rmpA, rmpD* and *rmpC*) or truncated as per inset legend.

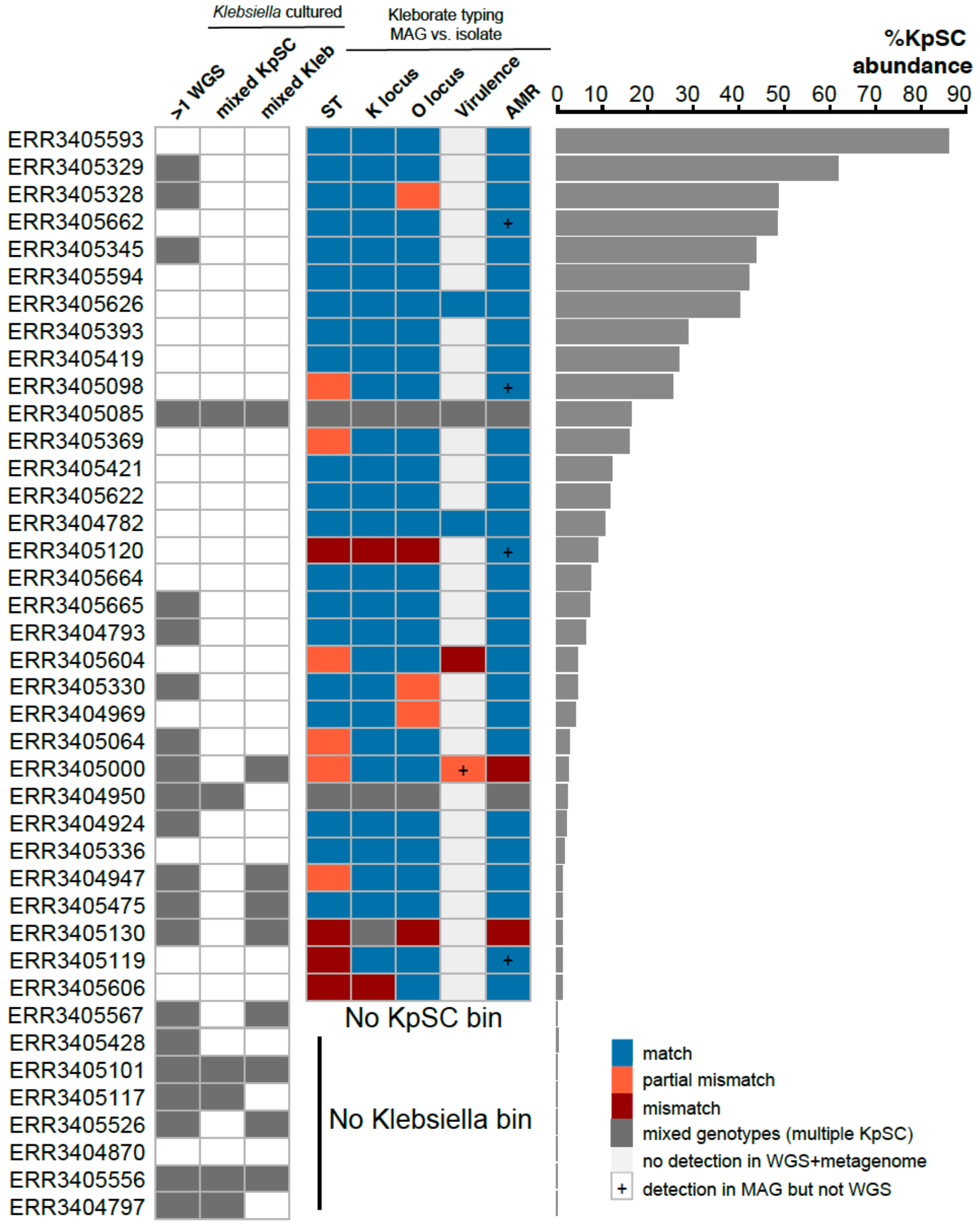

Supplementary Figure 9. Kleborate genotyping of metagenome-assembled genomes (MAGs) compared to whole genome sequences (WGS) of isolates cultured from the same samples, from the Baby Biome Study. Each row corresponds to one of 40 samples. First three columns indicate whether culture+WGS data provides evidence of (i) multiple KpSC strains, (ii) multiple KpSC species or (iii) multiple *Klebsiella* species (grey=yes, white=no). Subsequent columns indicate whether Kleborate genotyping of *Klebsiella*-assigned bins from MAGs matches genotyping of KpSC isolates cultured from the same

sample (colours as per legend). Barplots indicate %read abundance of KpSC in the metagenome sample. See Supplementary Data 8 and Supplementary Data 9 for more details. *K. pneumoniae* isolated but no KpSC-assigned MAG (estimated 0.16% relative abundance); *K. grimontii* isolated and MAG obtained (estimated 18.83% relative abundance).

Supplementary References

1. Brisse, S., Passet, V. & Grimont, P. A. D. Description of *Klebsiella quasipneumoniae* sp. nov., isolated from human infections, with two subspecies, *Klebsiella quasipneumoniae* subsp. *quasipneumoniae* subsp. nov. and *Klebsiella quasipneumoniae* subsp. *similipneumoniae* subsp. nov., and demonstration that Klebsiella singaporensis is a junior heterotypic synonym of *Klebsiella variicola*. *Int. J. Syst. Evol. Microbiol.* **64**, 3146–3152 (2014).
2. Rosenblueth, M., Martínez, L., Silva, J. & Martínez-Romero, E. *Klebsiella variicola*, A Novel Species with Clinical and Plant-Associated Isolates. *Syst. Appl. Microbiol.* **27**, 27–35 (2004).
3. Rodrigues, C. *et al.* Description of *Klebsiella africanensis* sp. nov., *Klebsiella variicola* subsp. *tropicalensis* subsp. nov. and *Klebsiella variicola* subsp. *variicola* subsp. nov. *Res. Microbiol.* **170**, 165–170 (2019).
4. Long, S. W. *et al.* Whole-Genome Sequencing of a Human Clinical Isolate of the Novel Species *Klebsiella quasivariicola* sp. nov. *Genome Announc.* **5**, e01057-17 (2017).
5. Lefort, V., Desper, R. & Gascuel, O. FastME 2.0: A Comprehensive, Accurate, and Fast Distance-Based Phylogeny Inference Program. *Mol. Biol. Evol.* **32**, 2798–2800 (2015).
6. Brisse, S. *et al.* Virulent clones of *Klebsiella pneumoniae*: Identification and evolutionary scenario based on genomic and phenotypic characterization. *PLoS One* **4**,

(2009).

7. Long, S. W. *et al.* Whole-genome sequencing of human clinical *Klebsiella pneumoniae* isolates reveals misidentification and misunderstandings of *Klebsiella pneumoniae, Klebsiella variicola*, and *Klebsiella quasipneumoniae*. *mSphere* **2**, e00290-17 (2017).
8. David, S. *et al.* Epidemic of carbapenem-resistant *Klebsiella pneumoniae* in Europe is driven by nosocomial spread. *Nat. Microbiol.* (2019). doi:10.1038/s41564-019-0492-8
9. Doyle, R. M. *et al.* Discordant bioinformatic predictions of antimicrobial resistance from whole-genome sequencing data of bacterial isolates: an inter-laboratory study. *Microb. genomics* **6**, e000335 (2020).
10. Neubauer, S. *et al.* A Genotype-Phenotype Correlation Study of SHV β-Lactamases Offers New Insight into SHV Resistance Profiles. *Antimicrob. Agents Chemother.* **64**, e02293-19 (2020).
11. Lin, T.-L. *et al.* Extended-Spectrum β-Lactamase Genes of *Klebsiella pneumoniae* Strains in Taiwan: Recharacterization of SHV-27, SHV-41, and TEM-116. *Microb. Drug Resist.* **12**, 12–15 (2006).
12. Arpin, C. *et al.* SHV-16, a β-Lactamase with a Pentapeptide Duplication in the Omega Loop. *Antimicrob. Agents Chemother.* **45**, 2480 LP – 2485 (2001).
13. Ma, L. *et al.* Novel SHV-Derived Extended-Spectrum β-Lactamase, SHV-57, That Confers Resistance to Ceftazidime but Not Cefazolin. *Antimicrob. Agents Chemother.* **49**, 600 LP – 605 (2005).
14. Ling, B.-D. *et al.* Characterisation of a novel extended-spectrum β-lactamase, SHV-70, from a clinical isolate of *Enterobacter cloacae* in China. *Int. J. Antimicrob. Agents* **27**, 355–356 (2006).
15. Mazzariol, A., Roelofsen, E., Koncan, R., Voss, A. & Cornaglia, G. Detection of a New SHV-Type Extended-Spectrum β-Lactamase, SHV-31, in a *Klebsiella*

pneumoniae Strain Causing a Large Nosocomial Outbreak in The Netherlands. *Antimicrob. Agents Chemother.* **51**, 1082 LP – 1084 (2007).
