# Supplementary material for: A genomic surveillance framework and genotyping tool for Klebsiella pneumoniae and its related species complex
Source: Nat Commun. 2021 Jul 7;12:4188. doi: 10.1038/s41467-021-24448-3 (PMC8263825; doi:10.1038/s41467-021-24448-3)
Supplement: Supplementary file 2 — Descriptions of Additional Supplementary Files [file 41467_2021_24448_MOESM2_ESM.pdf]

Descriptions of Additional Supplementary Files

Supplementary Data 1
Description: Kleborate species validation data.

Supplementary Data 2
Description: Isolate collection metadata and Kleborate genotyping results for publicly-available Klebsiella genomes analysed in this study.

Supplementary Data 3
Description: Comparison of virulence and AMR detection or genotypes by Kleborate vs. those reported in the EuSCAPE study.

Supplementary Data 4
Description: Data summary of publicly-available Klebsiella genomes.

Supplementary Data 5
Description: Virulence loci prevalence and lineage distribution in KpSC species.

Supplementary Data 6
Description: Frameshift mutations and incomplete loci detection in virulence determinants of non-redundant K. pneumoniae genomes.

Supplementary Data 7
Description: Summary of convergent genomes and convergence events.

Supplementary Data 8
Description: Kleborate genotyping results for metagenomes (non-binned vs. binned by species) and matched whole genome sequences from the Baby Biome Study.

Supplementary Data 9
Description: Summary of genotyping outputs for metagenomeassembled genomes (i.e. binned) vs. matched whole genome sequences from the Baby Biome Study.

Supplementary Data 10
Description: Studies citing Kleborate.

Supplementary Data 11
Description: Summary of key mutations in SHV and associations with β-lactamase subclasses.

Supplementary Data 12
Description: SHV mutations.

Supplementary Data 13
Description: Summary of publicly-available genome data.
