# Supplementary material for: A genomic surveillance framework and genotyping tool for Klebsiella pneumoniae and its related species complex
Source: Nat Commun. 2021 Jul 7;12:4188. doi: 10.1038/s41467-021-24448-3 (PMC8263825; doi:10.1038/s41467-021-24448-3)
Supplement: Supplementary file 6 — Supplementary data 4 [file 41467_2021_24448_MOESM6_ESM.docx]

**Supplementary Data 4. Data summary of publicly-available *Klebsiella* genomes**

Number of *Klebsiella* genomes (representative genomes indicated in brackets) included in study* by each of the categories specified

| **By species (subspecies)**** | | **By United Nations-defined region (countries)^#^** | | **By sample type^#^** | | **By year of collection^#^** | |
| --- | --- | --- | --- | --- | --- | --- | --- |
| **KpSC species**  *K. pneumoniae*  *K. quasipneumoniae*  (subsp. *quasipneumoniae*)  (subsp. *similipneumoniae*)  *K. variicola*  (subsp. *variicola*)  (subsp. *tropica*)  *K. quasivariicola*  *K. africana*  **Non-KpSC *Klebsiella* species**  *K. aerogenes*  *K. grimontii*  *K. huaxiensis*  *K. indica*  *K. michiganensis*  *K. oxytoca*  *K. pasteurii*  *K. spallanzanii*  **Unknown/**  **Non-*Klebsiella* species**  *Cedecea neteri*  *Enterobacter cloacae*  *E. coli/Shigella*  *Kosakonia cowanii*  *Serratia marcescens*  Unknown | **12493 (10720)**  11259 (9705)  163 (119)  490 (363)  541 (498)  23 (18)  16 (16)  1 (1)  **663 (557)**  244 (209)  106 (75)  4 (4)  2 (2)  171 (144)  106 (98)  26 (21)  4 (4)  **16 (NA)**  1  1  3  1  1  9 | **Africa (Northern)**  Egypt  **Africa (Sub-Saharan)**  Kenya  Madagascar  Malawi  Nigeria  Senegal  South Africa  Tanzania  **North America**  Canada  United States of America  **Latin America/Caribbean**  Brazil  Caribbean  Colombia  **Asia (East)**  China  Japan  South Korea  **Asia (Southeast)**  Cambodia  Laos  Singapore  Thailand  Vietnam  **Asia (South)**  India  Nepal  Pakistan  **Asia (West)**  Israel  Lebanon  Turkey  **Europe (East)**  Hungary  Poland  Romania  Russia  Slovakia  **Europe (North)**  Norway  United Kingdom  **Europe (South)**  Croatia  Greece  Italy  Portugal  Serbia  Spain  **Europe (West)**  Austria  Belgium  France  Germany  Netherlands  Switzerland  **Australia and New Zealand**  Australia  **Not specified** | **70 (67)**  35 (33)  **944 (865)**  183 (162)  265 (239)  71 (70)  128 (110)  138 (136)  86 (76)  49 (48)  **2171 (1994)**  44 (38)  2126 (1955)  **722 (684)**  238 (228)  248 (236)  191 (177)  **1693 (1330)**  1424 (1070)  153 (149)  48 (47)  **1223 (1009)**  247 (203)  95 (80)  77 (74)  552 (405)  182 (179)  **614 (513)**  207 (197)  166 (114)  210 (173)  **365 (333)**  107 (90)  80 (74)  170 (161)  **507 (482)**  48 (46)  128 (126)  141 (126)  111 (106)  40 (39)  **1306 (1047)**  54 (54)  1152 (896)  **1535 (1312)**  80 (78)  193 (167)  792 (615)  106 (98)  88 (83)  224 (220)  **780 (670)**  57 (44)  85 (84)  248 (231)  205 (149)  62 (48)  104 (98)  **588 (503)**  577 (492)  **633 (467)** | **Animals**  Bovine  Chicken  **Environmental**  Farm  Hospital/human-  associated habitat  Land/plant/vegetation  Sewage  Water  **Food**  Animal/animal product  Vegetation  **Human**  Abdominal  Abscess/fluid/pus  Blood/bone/sterile site  Catheter/tip/tube  External site  Faecal/intestinal/rectal  Liver  Mouth/nasal/throat  Lower Respiratory  Tissue  Urine/urogenital  Wound/trauma  **Lab strain**  **Other**  **Not specified** | **377 (272)**  102 (100)  64 (45)  **442 (372)**  25 (21)  98 (83)  62 (55)  100 (85)  78 (71)  **87 (79)**  48 (46)  35 (29)  **11704 (10214)**  94 (90)  280 (274)  2859 (2536)  102 (93)  48 (47)  1802 (1395)  174 (169)  66 (60)  1106 (993)  38 (38)  2191 (2034)  399 (389)  **54 (6)**  **12 (7)**  **480 (327)** | **1911-1949**  **1950-1979**  **1980-1989**  **1990-1999**  **2000-2009**  2000  2001  2002  2003  2004  2005  2006  2007  2008  2009  **2010-**  2010  2011  2012  2013  2014  2015  2016  2017  2018  2019  2020  **Not specified/**  **ambiguous** | **47 (36)**  **29 (27)**  **9 (9)**  **51 (51)**  **1278 (1228)**  15 (14)  53 (48)  94 (94)  68 (67)  109 (106)  171 (170)  190 (184)  166 (155)  194 (185)  218 (205)  **10477 (8897)**  260 (255)  432 (349)  750 (658)  2079 (1877)  2155 (1952)  1096 (846)  1652 (1372)  1203 (921)  694 (525)  152 (138)  4 (4)  **1265 (1029)** |

* Excluding n=336 genomes which failed assembly QC and n=163 duplicate assemblies (i.e. share the same biosample or strain ID as another genome)

**As detected by Kleborate

Only countries/samples with greater than 3 genomes are listed

# Excluding an additional n=14 genomes identified as unknown or species outside the *Klebsiella* genus
