# Supplementary material for: A genomic surveillance framework and genotyping tool for Klebsiella pneumoniae and its related species complex
Source: Nat Commun. 2021 Jul 7;12:4188. doi: 10.1038/s41467-021-24448-3 (PMC8263825; doi:10.1038/s41467-021-24448-3)
Supplement: Supplementary file 7 — Supplementary data 5 [file 41467_2021_24448_MOESM7_ESM.docx]

**Supplementary** **Data 5. Virulence loci prevalence and lineage distribution in *Klebsiella pneumoniae* species complex (KpSC) species**

| Virulence locus |  | *K. pneumoniae* | *K. quasipneumoniae subsp. quasipneumoniae* | *K. quasipneumoniae subsp.*  *similipneumoniae* | *K. quasivariicola* | *K. variicola subsp. tropica* | *K. variicola subsp. variicola* |
| --- | --- | --- | --- | --- | --- | --- | --- |
| Yersiniabactin (ybt) | **no ybt** | **6113** | **163** | **482** | **16** | **23** | **526** |
|  | **ybt** | **5146** | **0** | **8** | **0** | **0** | **15** |
|  | ybt0 | 138 | 0 | 0 | 0 | 0 | 2 |
|  | ybt1 | 255 | 0 | 0 | 0 | 0 | 0 |
|  | ybt2 | 67 | 0 | 0 | 0 | 0 | 1 |
|  | ybt3 | 9 | 0 | 0 | 0 | 0 | 0 |
|  | ybt4 | 103 | 0 | 1 | 0 | 0 | 4 |
|  | ybt5 | 41 | 0 | 0 | 0 | 0 | 0 |
|  | ybt6 | 16 | 0 | 0 | 0 | 0 | 0 |
|  | ybt7 | 3 | 0 | 1 | 0 | 0 | 0 |
|  | ybt8 | 45 | 0 | 0 | 0 | 0 | 0 |
|  | ybt9 | 1629 | 0 | 3 | 0 | 0 | 0 |
|  | ybt10 | 861 | 0 | 1 | 0 | 0 | 0 |
|  | ybt11 | 5 | 0 | 0 | 0 | 0 | 0 |
|  | ybt12 | 38 | 0 | 0 | 0 | 0 | 0 |
|  | ybt13 | 328 | 0 | 0 | 0 | 0 | 0 |
|  | ybt14 | 472 | 0 | 0 | 0 | 0 | 0 |
|  | ybt15 | 249 | 0 | 0 | 0 | 0 | 1 |
|  | ybt16 | 305 | 0 | 0 | 0 | 0 | 0 |
|  | ybt17 | 516 | 0 | 0 | 0 | 0 | 0 |
|  | ybt unknown | 66 | 0 | 1 | 0 | 0 | 7 |
| Colibactin  (clb) | **clb** | **872** | **0** | **0** | **0** | **0** | **0** |
|  | **no clb** | **10387** | **163** | **490** | **16** | **23** | **541** |
|  | clb 1 | 38 | 0 | 0 | 0 | 0 | 0 |
|  | clb 2 | 255 | 0 | 0 | 0 | 0 | 0 |
|  | clb 3 | 574 | 0 | 0 | 0 | 0 | 0 |
|  | clb unknown | 5 | 0 | 0 | 0 | 0 | 0 |
| Aerobactin  (iuc) | **iuc** | **1244** | **0** | **6** | **0** | **0** | **4** |
|  | **no iuc** | **10015** | **163** | **484** | **16** | **23** | **537** |
|  | iuc 1 | 924 | 0 | 4 | 0 | 0 | 2 |
|  | iuc 2 | 35 | 0 | 0 | 0 | 0 | 0 |
|  | iuc 2A | 60 | 0 | 0 | 0 | 0 | 0 |
|  | iuc 3 | 115 | 0 | 2 | 0 | 0 | 0 |
|  | iuc 4 | 6 | 0 | 0 | 0 | 0 | 0 |
|  | iuc 5 | 13 | 0 | 0 | 0 | 0 | 2 |
|  | iuc unknown | 90 | 0 | 0 | 0 | 0 | 0 |
|  | iuc1 iuc3 | 1 | 0 | 0 | 0 | 0 | 0 |
| Salmochelin  (iro) | **iro** | **725** | **0** | **4** | **0** | **0** | **5** |
|  | **no iro** | **10534** | **163** | **486** | **16** | **23** | **536** |
|  | iro 1 | 578 | 0 | 4 | 0 | 0 | 3 |
|  | iro 2 | 37 | 0 | 0 | 0 | 0 | 0 |
|  | iro 3 | 59 | 0 | 0 | 0 | 0 | 1 |
|  | iro 4 | 4 | 0 | 0 | 0 | 0 | 0 |
|  | iro 5 | 6 | 0 | 0 | 0 | 0 | 0 |
|  | iro unknown | 39 | 0 | 0 | 0 | 0 | 1 |
|  | iro1 iro3 | 2 | 0 | 0 | 0 | 0 | 0 |
| Hypermucoidy rmp (rmpADC) | **rmp** | **867** | **0** | **4** | **0** | **0** | **4** |
|  | **no rmp** | **10392** | **163** | **486** | **16** | **23** | **537** |
|  | rmp 1 | 684 | 0 | 4 | 0 | 0 | 3 |
|  | rmp 2 | 32 | 0 | 0 | 0 | 0 | 0 |
|  | rmp 2A | 54 | 0 | 0 | 0 | 0 | 0 |
|  | rmp 3 | 54 | 0 | 0 | 0 | 0 | 1 |
|  | rmp unknown | 34 | 0 | 0 | 0 | 0 | 0 |
|  | rmp1 rmp3 | 9 | 0 | 0 | 0 | 0 | 0 |
| Hypermucoidy *rmpA2* gene | rmpA2 | **812** | **0** | **3** | **0** | **0** | **2** |
|  | no rmpA2 | **10447** | **163** | **487** | **16** | **23** | **539** |
|  | truncated rmpA2 | 717 | 0 | 3 | 0 | 0 | 2 |
|  | intact rmpA2 | 95 | 0 | 0 | 0 | 0 | 0 |
