# Supplementary material for: A genomic surveillance framework and genotyping tool for Klebsiella pneumoniae and its related species complex
Source: Nat Commun. 2021 Jul 7;12:4188. doi: 10.1038/s41467-021-24448-3 (PMC8263825; doi:10.1038/s41467-021-24448-3)
Supplement: Supplementary file 8 — Supplementary data 6 [file 41467_2021_24448_MOESM8_ESM.docx]

**Supplementary Data 6. Frameshift mutations and incomplete loci detection in virulence determinants of non-redundant *K. pneumoniae* genomes**

|  | Number of non-redundant *K. pneumoniae* genomes | | | | | |
| --- | --- | --- | --- | --- | --- | --- |
| Virulence locus | With virulence locus | Intact | Non-intact | Truncated i.e. frameshift mutations) | Incomplete i.e. missing gene(s) | Truncated + incomplete |
| Yersiniabactin | 4309 | 3877 | 432 | 375 | 44 | 13 |
| Colibactin | 794 | 568 | 226 | 190 | 18 | 18 |
| Aerobactin | 1090 | 942 | 148 | 130 | 14 | 4 |
| Salmochelin | 683 | 562 | 121 | 72 | 21 | 28 |
| Rmp | 782 | 552 | 230 | 184 | 39 | 7 |
