# Supplementary material for: A genomic surveillance framework and genotyping tool for Klebsiella pneumoniae and its related species complex
Source: Nat Commun. 2021 Jul 7;12:4188. doi: 10.1038/s41467-021-24448-3 (PMC8263825; doi:10.1038/s41467-021-24448-3)
Supplement: Supplementary file 13 — Supplementary data 11 [file 41467_2021_24448_MOESM13_ESM.docx]

**Supplementary Data 11. Summary of key mutations in SHV and associations with β-lactamase subclasses**

| AA site (Ambler) | Residue in SHV-1 | Known mutations | Class modifying | >2x increase MIC# |
| --- | --- | --- | --- | --- |
| 238 | G | S, A | ESBL | CTX, CRO, CPD, FEP, CPT, ATM |
| 179 | D | N, A, G | ESBL | CTX, CRO, CAZ, FEB [only tested for 179G] |
| 148 | L | V | ESBL (only with 35Q mutation) | CPT |
| 164-179 (omega loop) | RWETELNEALPGDARD | Any change | ESBL | - |
| 69 | M | I, L | β-lactamase inhibitor | TAZ |
| 234 | K | R | β-lactamase inhibitor | AVI |
| 235 | T | A | β-lactamase inhibitor | - |
| 25 | A | T, S | - | CPT |
| 35 | L | Q | - | CPT |
| 156 | G | D | - | CPT |
| 146 | A | T, V | - | CPT |
| 240 | E | K, R | - | CPT, TAZ |

**Notes**

# as detected in *E. coli* construct in Neubauer 2020 (Antimicrob. Agents Chemother 64:e02293-19)

**Abbreviations:**

AA site, amino acid site

CTX, cefotaxime (third-generation cephalosporin)

CRO, ceftriaxone (third-generation cephalosporin)

CPD, cefpodoxime (third-generation cephalosporin)

CAZ, ceftazidime (third-generation cephalosporin)

FEP, cefepime (fourth-generation cephalosporin)

CPT, ceftaroline (fifth-generation cephalosporin)

ATM, aztreonam (monobactam)

TAZ, tazobactam (β-lactamase inhibitor)

AVI, avibactam (β-lactamase inhibitor)
