# Supplementary material for: A genomic surveillance framework and genotyping tool for Klebsiella pneumoniae and its related species complex
Source: Nat Commun. 2021 Jul 7;12:4188. doi: 10.1038/s41467-021-24448-3 (PMC8263825; doi:10.1038/s41467-021-24448-3)
Supplement: Supplementary file 15 — Supplementary data 13 [file 41467_2021_24448_MOESM15_ESM.docx]

**Supplementary Data 13. Summary of publicly-available genome data***

| **Database/study** | **Number of genomes** | **Notes** | **Reference** |
| --- | --- | --- | --- |
| NCBI RefSeq | 10500 |  | All assemblies assigned to the *Klebsiella* genus available on NCBI as of July 17^th^ 2020. |
| **Assembled from reads** | | | |
| Alice Springs | 18 | Isolates from Australia | Wyres et al. 2016^1^ |
| Bialek 2014 | 38 | Isolates from multiple countries and sources | Bialek-Davenet et al. 2014^2^ |
| Bowers 2015 | 115 | Isolates from multiple countries and sources | Bowers et al. PLoS One 2015^3^ |
| Chung The 2015 | 75 | Isolates from outbreak, Patan Hospital, Nepal | Chung The et al. 2015^4^ |
| Davis 2015 | 59 | Isolates from retail meats and UTIs of patients, Flagstaff Medical Center, Arizona, USA | Davis et al. 2015^5^ |
| DeLeo 2014 | 78 | Isolates from multiple countries and sources | DeLeo et al. 2014^6^ |
| Ellington 2019 | 177 | Infection isolates from patients, Cambridge University Hospitals NHS Foundation Trust, UK | Ellington et al. 2019^7^ |
| Gorrie 2017/2018 | 424 | Carriage and infection isolates from patients, Alfred Hospital Network, Australia | Gorrie et al. 2017^8^  Gorrie et al. 2018^9^ |
| Henson 2017 | 183 (+X non Kp) | Blood culture isolates from patients, Kilifi County Hospital, Kenya | Henson et al. 2017^10^ |
| Holt 2015 | 250 | Isolates from multiple countries and sources | Holt et al. 2015^11^ |
| Huynh 2020 | 490 | Carriage isolates from pregnant women in Madagascar, Cambodia and Senegal | Huynh et al. 2020^12^ |
| Lee 2016 | 26 | Isolates from ‘Antibiotics for Klebsiella Liver Abscess Syndrome Study’, Singapore | Lee et al. 2016^13^ |
| Musicha 2019 | 71 | Invasive and carriage isolates from patients, Queen Elizabeth Central Hospital, Malawi | Musicha et al. 2019^14^ |
| Smit 2018 | 90 | Isolates from patients, Angkor Hospital for Children, and environmental sites, Cambodia | Smit et al. 2018^15^ |
| Stoesser 2013 | 69 | Blood culture isolates from patients, Oxford University Hospitals NHS Trust, UK | Stoesser et al. 2013^16^ |
| Stoesser 2014 | 53 | Invasive isolates from neonatal outbreak, Patan Hospital, Nepal | Stoesser et al. 2014^17^ |
| Struve 2015 | 53 | Isolates from multiple countries and sources | Struve et al. 2015^18^ |
| Wand 2016 | 24 | Isolates collected from the pre-antibiotic era | Wand et al. 2016^19^ |
| Wyres 2020 | 363 | Blood culture isolates from South and Southeast Asia | Wyres et al. 2020^20^ |

*Note. numbers exclude n=295 genomes which failed assembly QC and n=163 duplicate samples (i.e. identical biosample numbers present in multiple datasets/studies)

**References**

1. Wyres, K. L. *et al.* Identification of *Klebsiella* capsule synthesis loci from whole genome data. *Microb. Genomics.* **2**, (2016).

2. Bialek-davenet, S. *et al.* Genomic Definition of Hypervirulent and Multidrug-Resistant *Klebsiella pneumoniae* Clonal Groups. *Emerg. Infect. Dis.* **20**, 1812–1820 (2014).

3. Bowers, J. R. *et al.* Genomic analysis of the emergence and rapid global dissemination of the clonal group 258 *Klebsiella pneumoniae* pandemic. *PLoS One* **10**, 1–24 (2015).

4. Chung The, H. *et al.* A high-resolution genomic analysis of multidrug-resistant hospital outbreaks of *Klebsiella pneumoniae*. *EMBO Mol. Med.* **7**, 227–239 (2015).

5. Davis, G. S. *et al.* Intermingled *Klebsiella pneumoniae* populations between retail meats and human urinary tract infections. *Clin Infect Dis* **61**, 892–899 (2015).

6. Deleo, F. R. *et al.* Molecular dissection of the evolution of carbapenem-resistant multilocus sequence type 258 *Klebsiella pneumoniae*. *Proc. Natl. Acad. Sci. U. S. A.* **111**, 4988–4993 (2014).

7. Ellington, M. J. *et al.* Contrasting patterns of longitudinal population dynamics and antimicrobial resistance mechanisms in two priority bacterial pathogens over 7 years in a single center. *Genome Biol.* **20**, 184 (2019).

8. Gorrie, C. L. *et al.* Gastrointestinal carriage is a major reservoir of *K. pneumoniae* infection in intensive care patients. *Clin Infect Dis* **65**, 208–215 (2017).

9. Gorrie, C. L. *et al.* Antimicrobial resistant *Klebsiella* *pneumoniae* carriage and infection in specialized geriatric care wards linked to acquisition in the referring hospital. *Clin. Infect. Dis.* **67**, 161–170 (2018).

10. Henson, S. P. *et al.* Molecular epidemiology of *Klebsiella* *pneumoniae* invasive infections over a decade at Kilifi County Hospital in Kenya. *Int. J. Med. Microbiol.* **307**, 422–429 (2017).

11. Holt, K. E. *et al.* Genomic analysis of diversity, population structure, virulence, and antimicrobial resistance in *Klebsiella pneumoniae*, an urgent threat to public health. *Proc Natl Acad Sci USA* **112**, E3574--81 (2015).

12. Huynh, B.-T. *et al.* *Klebsiella* *pneumoniae* carriage in low-income countries: antimicrobial resistance, genomic diversity and risk factors. *Gut Microbes* **11**, 1287–1299 (2020).

13. Lee, I. R. *et al.* Differential host susceptibility and bacterial virulence factors driving *Klebsiella* liver abscess in an ethnically diverse population. *Sci Rep* **6**, 29316 (2016).

14. Musicha, P. *et al.* Genomic analysis of *Klebsiella* *pneumoniae* isolates from Malawi reveals acquisition of multiple ESBL determinants across diverse lineages. *J. Antimicrob. Chemother.* **74**, 1223–1232 (2019).

15. Smit, P. *et al.* Transmission dynamics of hyper-endemic multi-drug resistant *Klebsiella* *pneumoniae* in a Southeast Asian neonatal unit: a longitudinal study with whole genome sequencing. *Front Microbiol.* **9**, 1197 (2018).

16. Stoesser, N. *et al.* Predicting antimicrobial susceptibilities for *Escherichia coli*and *Klebsiella pneumoniae* isolates using whole genomic sequence data. *J. Antimicrob. Chemother.* **68**, 2234–2244 (2013).

17. Stoesser, N. *et al.* Genome sequencing of an extended series of NDM-producing *Klebsiella pneumoniae* isolates from neonatal infections in a Nepali hospital characterizes the extent of community- Versus hospital- associated transmission in an endemic setting. *Antimicrob. Agents Chemother.* **58**, 7347–7357 (2014).

18. Struve, C. *et al.* Mapping the evolution of hypervirulent *Klebsiella pneumoniae*. *MBio* **6**, 1–12 (2015).

19. Wand, M. E. *et al.* Characterization of pre-antibiotic era *Klebsiella pneumoniae* isolates with respect to antibiotic/disinfectant susceptibility and virulence in *Galleria mellonella*. *Antimicrob. Agents Chemother.* **59**, 3966–3972 (2015).

20. Wyres, K. L. *et al.* Genomic surveillance for hypervirulence and multi-drug resistance in invasive *Klebsiella* *pneumoniae* from South and Southeast Asia. *Genome Med.* **12**, 11 (2020).
