# Supplementary material for: A genomic surveillance framework and genotyping tool for Klebsiella pneumoniae and its related species complex
Source: Nat Commun. 2021 Jul 7;12:4188. doi: 10.1038/s41467-021-24448-3 (PMC8263825; doi:10.1038/s41467-021-24448-3)
Supplement: Supplementary file 16 — Reporting Summary [file 41467_2021_24448_MOESM16_ESM.pdf]

nature research

Corresponding author(s): Margaret Lam

Last updated by author(s): Jun 1, 2021

Reporting Summary

Nature Research wishes to improve the reproducibility of the work that we publish. This form provides structure for consistency and transparency in reporting. For further information on Nature Research policies, see our Editorial Policies and the Editorial Policy Checklist.

Statistics

For all statistical analyses, confirm that the following items are present in the figure legend, table legend, main text, or Methods section.

n/a	Confirmed	
☐	☒	The exact sample size (n) for each experimental group/condition, given as a discrete number and unit of measurement
☒	☐	A statement on whether measurements were taken from distinct samples or whether the same sample was measured repeatedly
☐	☒	The statistical test(s) used AND whether they are one- or two-sided<br>*Only common tests should be described solely by name; describe more complex techniques in the Methods section.*
☒	☐	A description of all covariates tested
☒	☐	A description of any assumptions or corrections, such as tests of normality and adjustment for multiple comparisons
☐	☒	A full description of the statistical parameters including central tendency (e.g. means) or other basic estimates (e.g. regression coefficient) AND variation (e.g. standard deviation) or associated estimates of uncertainty (e.g. confidence intervals)
☐	☒	For null hypothesis testing, the test statistic (e.g. F, t, r) with confidence intervals, effect sizes, degrees of freedom and P value noted<br>*Give P values as exact values whenever suitable.*
☒	☐	For Bayesian analysis, information on the choice of priors and Markov chain Monte Carlo settings
☒	☐	For hierarchical and complex designs, identification of the appropriate level for tests and full reporting of outcomes
☐	☒	Estimates of effect sizes (e.g. Cohen's d, Pearson's r), indicating how they were calculated

Our web collection on statistics for biologists contains articles on many of the points above.

Software and code

Policy information about availability of computer code

Data collection	No software was used for data collection
Data analysis	Kleborate v.2.0.0 (https://github.com/katholt/Kleborate/)<br>Mash v2.1<br>Unicycler v0.4.7<br>SPAdes v 3.13.1<br>MaxBin v2.2.7<br>Kraken v2.0.7<br>Bracken v2.5<br>R v1.1.456<br>ggplot v3.2.0<br>pheatmap v1.0.12<br>Microreact v102.0.0 (www.microreact.org)

For manuscripts utilizing custom algorithms or software that are central to the research but not yet described in published literature, software must be made available to editors and reviewers. We strongly encourage code deposition in a community repository (e.g. GitHub). See the Nature Research guidelines for submitting code & software for further information.

Data

Policy information about availability of data

All manuscripts must include a data availability statement. This statement should provide the following information, where applicable:

- Accession codes, unique identifiers, or web links for publicly available datasets
- A list of figures that have associated raw data
- A description of any restrictions on data availability

Supplementary Data 2 lists accession numbers for each genome analyzed in this study, alongside the isolate collection metadata where available and Kleborate-generated output. An interactive phylogeny of all public Klebsiella genomes alongside (i) the corresponding Kleborate data or (ii) information relating to convergence events can be accessed at (i) http://microreact.org/project/bQmTJfQmiCpFBjhoacaL8u and (ii) https://microreact.org/project/JDyan46yctyDh6weEUjWN respectively. Supplementary Data 8 lists accession numbers for metagenome reads, matched isolate whole genome assemblies and the Kleborate-generated output.

Field-specific reporting

Please select the one below that is the best fit for your research. If you are not sure, read the appropriate sections before making your selection.

☐ Life sciences ☐ Behavioural & social sciences ☒ Ecological, evolutionary & environmental sciences

For a reference copy of the document with all sections, see nature.com/documents/nr-reporting-summary-flat.pdf

Ecological, evolutionary & environmental sciences study design

All studies must disclose on these points even when the disclosure is negative.

Study description	Application of the genotyping tool Kleborate on 13,156 publicly available Klebsiella genomes, and metagenome-assembled assemblies and matched isolate whole genome sequences from 40 gut metagenome samples from the Baby Biome Study (Shao et al. 2019)
Research sample	(1) 13,156 publicly available Klebsiella genomes collected from 99 countries and between 1920-2020 based on available metadata. (2) n=47 metagenome samples reporting KpSC detection from the Baby Biome Study with matched isolate whole genome sequencing (Shao et al. 2019)
Sampling strategy	All publicly available Klebsiella whole genome sequences available at the time of the study were included.
Data collection	A summary of the sources from which the 13,156 Klebsiella genomes were collected is provided in Supplementary Data 13, and includes published studies from which reads were downloaded and then assembled, and all Klebsiella assemblies deposited in RefSeq as of July 17 2020. The genomes from published studies have been downloaded and curated (i.e. subjected to read/assembly QC) by Lam M.M.C and Wyres K.L. Reads and isolate whole genome sequences from the Baby Biome Study were downloaded using the accession numbers provided in Shao et al's study, and also listed in Supplementary Data 8.
Timing and spatial scale	Based on samples with metadata provided (i.e. excluding samples with unknown collection dates, country of isolation etc), the genomes are derived from isolates collected in 99 countries across Oceania, Asia, Europe, Americas, and Africa, and between 1920-2020. The Baby Biome Study included gut metagenome samples collected from mothers or infants in the UK between May 2014 and December 2017 (Shao et al. 2019)
Data exclusions	An additional 499 genomes were excluded due to data redundancy (i.e. only a single genome corresponding to each unique biosample identifier was included for analysis) or failing to meet assembly QC metrics (as detailed in Methods). These genomes are listed in the 'Excluded genomes' table of Supplementary Data 2.<br>Of the 47 metagenome samples (Shao et al. 2019), 7 did not assemble due to memory and compute walltime constraints, and were therefore excluded.
Reproducibility	To ensure reproducibility, the Kleborate tool has been tested by multiple users. Genotyping results generated from Kleborate for a test set were also independently verified using BLAST, which yielded concordant results.
Randomization	Randomization was not applicable to this study as the research sample encompassed all publicly available whole genome data at the time of the study.
Blinding	As the research sample encompassed all publicly available whole genome data, any potential sources of bias that could be accounted for by blinding was irrelevant.

Did the study involve field work? ☐ Yes ☒ No

Reporting for specific materials, systems and methods

We require information from authors about some types of materials, experimental systems and methods used in many studies. Here, indicate whether each material, system or method listed is relevant to your study. If you are not sure if a list item applies to your research, read the appropriate section before selecting a response.

Materials & experimental systems

n/a	Involved in the study
☒	☐ Antibodies
☒	☐ Eukaryotic cell lines
☒	☐ Palaeontology and archaeology
☒	☐ Animals and other organisms
☒	☐ Human research participants
☒	☐ Clinical data
☒	☐ Dual use research of concern

Methods

n/a	Involved in the study
☒	☐ ChIP-seq
☒	☐ Flow cytometry
☒	☐ MRI-based neuroimaging
